# Examining Health Inequalities in Latvia: A Decade of Association between Socioeconomic Position and Perceived Health Status

**DOI:** 10.1155/2017/7541416

**Published:** 2017-07-27

**Authors:** Anželika Berķe-Berga, Pavitra Paul, Hannu Valtonen

**Affiliations:** ^1^Department of Regional Economics and Business, Rīga Stradiņš University, Riga LV-1067, Latvia; ^2^Department of Health and Social Management, University of Eastern Finland, P.O. Box. 1627, 70211 Kuopio, Finland

## Abstract

The relationship between socioeconomic position (SEP) and population health is contextual. This study identifies the determinants of SEP producing health inequalities in the Latvian population. We also estimate the proportional contribution of different socioeconomic strata- (SES-) related determinants in Latvian health inequalities and measure the changes in the relative contributions of such determinants over the period 2005–2015. Using the household survey data (2005–2015), we construct a principal component analysis based SES index. A regression-based concentration index (CI) is our measure of health inequality to examine the distribution of perceived health status. Finally, we identify and estimate the contribution of predictors of health inequalities by decomposing CI with Oaxaca-Blinder decomposition. SES-related health inequalities have declined from 2005 (CI: 0.201) to 2015 (CI: 0.137) in Latvia—better-off Latvians enjoyed better perceived health during that period. The proportional contributions of education and working status have increased in 2015 compared to 2005. Although we have generated the first evidence to suggest policy relevant measures in addressing Latvian health inequalities, our decomposition method explains the extent of variation in perceived health instead of covariance between health and SEP.

## 1. Introduction

Health inequality is defined as differences in health among individuals or between groups (socioeconomic, geography, education, race, etc.). The linkage between increasing income inequality and the worsening of health status is well documented [1–7]. People who live in the lowest socioeconomic strata (SES) are vulnerable to ill health [8, 9]. Lochner et al. [10] have summarized evidence of a high risk of death when living in a high-inequality environment.

Less education, low income or unemployment, and lower position in the hierarchal society have a strong positive association with lower levels of perceived health [11]. Gilson [12], Wilkinson [13], Manor et al. [14], Mcisaac and Wilkinson [15], Kunst and Mackenbach [16], Blaxter [17], and Jones and Moon [18] conclude that in-country distribution of material deprivation reflects in-country health differences,* ceteris paribus*. Marmot et al. [19] have suggested that different mechanisms operate at the top and at the bottom of SES.

Eikemo et al. [20, 21] argue that although individual factors account for variations in health, welfare state arrangements is an important factor explaining variations in population health between countries. Rodgers [22] suggests that levels of health serve as a signal of the socioeconomic environment where people live and reflect the level of deprivation of the society. Research has established a strong association between socioeconomic position (SEP) and perceived health; however, such association is confounded by factors that are often not global, rather determined by socioeconomic and political contexts [23].

Economic development literatures advocate the recognition of health as a reflection of societal well-being, when the development is in transition [24, 25]. Furthermore, the concern for poverty and inequality necessitates the focus of interest not in health status that is apparent in the society as a whole but in the health status of different socioeconomic groups [26]. Socioeconomic inequalities in health are a major challenge for health policy effectiveness because a reduction in the burden of health problems in worse-off (more material deprivation) groups offers an enormous potential for improving the average health status of the population as a whole.

Research has found existence of health inequalities in central and eastern European societies [27, 28]. A few studies have also documented SES-related health differences in the Baltic Republics: Lithuania, Latvia, and Estonia [11, 29–31]. Monden [29] has further concluded that substantial inequalities in self-assessed health exist in the Baltic States and such inequalities were stable during the last few years of the last century. Monden [11] has demonstrated a relatively stronger effect of income and working status on perceived health compared to educational achievements for Latvians.

Following the disintegration of the USSR, the social and economic reforms that are taking place in Latvia are likely to have an effect on daily life [32–34]. The accession of Latvia to the European Union in 2004 has triggered further a new phase of development but did not end the ongoing social reforms. The distributions of the determinants of perceived health status are time variant phenomena. So, the importance of time and timing in understanding the causal links between exposures and outcomes cannot be ignored. Material factors are linked to conditions of economic hardship, as well as to health damaging conditions in the physical environment (e.g., housing conditions, physical working environment, etc.). Thus, health inequalities result from the differential accumulation of exposures and experiences that have their sources in the material world.

Unlike financial resources which can be equalized over time with the payment of interests, developmental inputs are not necessarily fungible. A clear gradient exists in the effect of exposure to disadvantaged SEP on health; the extent of health risk increases with each additional level of exposure [35]. Using a long-run panel (1994–2013) from waves of Russian Longitudinal Monitoring Survey datasets, Paul and Valtonen [36] infer that SES specific mean and the distribution of perceived health status within SES are important guides to improve average health of the Russian population. Although the lasting effect of transitional changes on the Latvian population health is an obvious phenomenon, the evidence is limited [37, 38].

This study is to examine health inequalities in Latvia and identify the determinants of SEP producing such health inequalities in the Latvian population.

We

1. examine the distribution of perceived health across Latvians of different socioeconomic groups;
2. estimate the proportional contribution of different SES-related determinants in Latvian health inequalities;
3. measure the changes in the relative contributions of such determinants over the period 2005–2015.

## 2. Materials and Methods

We used data from 11 (2005–2015) waves of household survey (Latvian Statistical Bureau: http://www.csb.gov.lv/en/dati/statistics-database-30501.html). This annual survey contains an array of information on the economic, social, demographic, and health characteristics of respondents, their households, and the environments where they live. Following accession to the EU in 2004, the survey design, stratification, and sampling units remained consistent from 2005 onwards. The survey uses weights to account for nonresponse and attrition. Our data (Table 1) was cross-sectional time series with a total of 124,934 respondents nested in 6,599 households.

Our dependent variable for the analysis was perceived (self-assessed) health (SAH). Individuals were asked, “How would you evaluate your health?” And the response was recorded on a five-point Likert scale with the answers “very good,” “good,” “average—not good but not bad,” “bad,” and “very bad.” SAH variables have been widely used in literatures [39–42] that analyze the socioeconomic health gradient.

SES is a multifaceted concept; no direct measure is available. Heterogeneity in relevant individual and household circumstances, intertemporal consumption smoothing, and interpersonal income sharing entail that neither measured current income (it is instructive that, in the same setting, Ravallion and Loskhin (2001) find evidence that many “nonincome” factors at the individual and household levels impinge on perceived economic welfare in Russia at given current incomes or expenditures on consumption deflated by standard poverty lines) nor consumption (there are uncertainties about how to best normalize for heterogeneity in consumption needs, such as stemming from demographic differences between households (Pollak, 1991); for example, the poverty lines used as deflators may not correctly weight differences in household size or demographic composition) is a particularly good proxy for economic welfare, as relevant to perceived health status. Principal component analysis works on the covariance or correlation matrix to extract the directions in the multivariate space that is the “most informative,” that is, reflecting the greatest variability.

We used adult equivalent household income (the household income was deflated to the value of 2005; we calibrated the household income as per adult equivalent using the modified OECD scale; the Statistical Office of the European Union (EUROSTAT) adopted in the late 1990s the so-called “OECD-modified equivalence scale”; this scale, first proposed by Haagenars et al. (1994), assigns a value of 1 to the household head, of 0.5 to each additional adult member, and of 0.3 to each child), working status, level of education, ownership of fixed assets (ownership of house), ownership of durable assets (washing machine, computer, and car), available floor space in square meter for living, and living standards (condition of dwelling unit and availability of enough heating provision in the household) to arrive at weights for the proxies of material affluence. The inclusion of a sufficiently broad range of variables and also a continuous variable (available floor space in square meter) enabled us to construct the SES indices without the problems of truncation (truncation implies even distribution of SES spread over a narrow range, which makes differentiation between the SES difficult) [43]. We validated sampling adequacy for the variables used by Kaiser–Meyer–Olkin score (0.80 and above). We used the weighted sum of standardized variables to obtain the SES score. Finally, households were grouped into SES quintiles. We also measured inequality in income by the Gini index ($G=2\mathrm{covar}\left(y,r_{y}\right)/N\overset{-}{y}$, where covar⁡(*y*, *r*_*y*_) is the covariance between income (*y*) and ranks of all households according to the income (*r*_*y*_) ranging from the poorest household (rank = 1) to the richest (rank  =  *N*), *N* is the total number of households, and $\overset{-}{y}$ is the mean of the adult equivalent household income (YitzhakI, 1994; Lerman and Yitzhaki, 1984)).

We standardized [44, 45] perceived health status applying an indirect method of standardization (indirectly standardized health is the difference between observed and expected health where expected health for an individual is the average health of individuals with the same levels of standardizing variables as the individual; with groups, expected health for an SES group is the weighted average of health levels conditional on the standardizing variables, where the weights are the proportion of the SES group population in the subgroups defined by the standardizing variables). We estimated the correlation of confounding variables (age, gender, diagnosed chronic diseases, and presence of physical limitation) with perceived health status conditional on nonconfounding variables (region and SES). This regression-based approach (see (1)) “corrects” the actual distribution of perceived health status by comparing it with the distribution that would be observed if all individuals in the group had their own age, gender, diagnosed chronic diseases, and presence of physical limitation characteristics but the same mean age, gender, diagnosed chronic diseases, and presence of physical limitation effect as the entire population.

(1) $$\begin{array}{c} y_{i}=\alpha +\underset{j}{\sum }\beta_{j}x_{ji}+\underset{k}{\sum }\gamma_{k}z_{ki}+\epsilon_{i}, \end{array}$$

where *y*_*i*_ is perceived health status; *i* denotes the individual; and *α*, *β*, and *γ* are parameter vectors. *x*_*j*_ are confounding variables (age, gender, diagnosed chronic diseases, and presence of physical limitation), which we standardize, and *z*_*k*_ are nonconfounding variables (region and SES), which we do not standardize but control for in order to estimate partial correlations with the confounding variables. The Newey-West (a regression method that corrects for heteroskedasticity and autocorrelation) estimator estimates ($\hat{\alpha }{,\hat{\beta }}_{j},{\hat{\gamma }}_{k}$) the individual values of the confounding variables (*x*_*ji*_), and sample means of the nonconfounding variables (${\overset{-}{z}}_{k}$) are then used to obtain the predicted, or “*x*-expected,” values of the perceived health status ${\hat{y}}_{i}^{x}$:

(2) $$\begin{array}{c} {\hat{y}}_{i}^{x}=\hat{\alpha }+\underset{j}{\sum }{\hat{\beta }}_{j}x_{ji}+\underset{k}{\sum }{\hat{\gamma }}_{k}{\overset{-}{z}}_{k}. \end{array}$$

Estimates of indirectly standardized perceived health are

(3) $$\begin{array}{c} {\hat{Y}}_{i}^{IS}=Y_{i}-{\hat{Y}}_{i}^{X}+\overset{-}{Y}, \end{array}$$

where

- ${\hat{Y}}_{i}^{IS}$ is indirectly standardized, perceived health status;
- *Y*_*i*_ is actual health;
- ${\hat{Y}}_{i}^{X}$ is *x*-expected health;
- $\bar{Y}$ is overall sample mean.

In the next step, following the principles of previous analyses [46, 47], we dichotomized the five-scaled measure into a binary variable [48], “perceived health” (1 = good, i.e., responded as “very good,” “good,” and “average”; 0 = not good, i.e., responded as “bad” and “very bad”).

The conventional regression-based statistical methods report the magnitude and the direction of association between SEP and health status of the individual but ignore possibility of variance in the effect of explanatory variables across distribution. Further, such traditional methods cannot reflect the extent of health differences across SES of the population and thus do not allow for comparison over time [46]. Therefore, we used the health concentration index (CI) as our measure of SES-related inequality.

The concentration curve plots the cumulative proportion of perceived health (*y*) against the cumulative share of the population ranked by SES variables. The curve lies below the 45° line (diagonal) of equality, if perceived health is concentrated among the better-off and above the 45° line (diagonal) of equality, if perceived health is concentrated among the worse-off. The CI is defined as twice the area between the concentration curve and the diagonal (the line of equality):

(4) $$\begin{array}{c} CI=\frac{2}{n\mu }\overset{n}{\underset{i=1}{\sum }}y_{i}R_{i}-1, \end{array}$$

where *n* is the sample size and *R* denotes the individual's fractional rank (position of the individual) in the SES distribution. *μ* is the mean of the binary variable *y* (perceived health status) whose distribution across SES is the subject of interest. For *μ* > 0 (if *y* = 0 for all *i*, CI is undefined), the minimum value of CI is equal to *μ* − 1 + (1/*n*), and the maximum value is equal to 1 − *μ* + (1/*n*).

For a given *μ* > 0, the maximum of the CI is when the poorest *j* individuals have a value of *y* equal to zero, and the richest *n* − *j* individuals have a value of *y* equal to one.

Therefore, *μ* = (*n* − *j*)/*n* and CI = 1 − *μ* + 1/*n*. For the large samples, the 1/*n* term vanishes, and the minimum and maximum tend to *μ* − 1 and 1 − *μ*, respectively [49]:

(5) $$\begin{array}{c} R_{i}=\overset{i-1}{\underset{j=1}{\sum }}w_{j}+\frac{1}{2}w_{i}, \end{array}$$

where *w*_0_ = 0. *R*_*i*_ denotes the weighted cumulative proportion of the population up to the midpoint of each individual weight and is bounded in the (0; 1) interval. *R*_*i*_ represents the cumulative distribution function of SES and indicates the individual's position within the SES distribution.

We estimated CI from regression of a transformation (correction of the standard error across SES correlation owing to the rank nature of the regressor) of the perceived health status on the fractional rank in SES distribution [50].

CI becomes positive if health (i.e., perceived health status) is concentrated among the better-off, negative if health (i.e., perceived health status) is concentrated among the worse-off, and zero if no inequality is observed. Thus, CI can also be interpreted as the slope of a line passing through the heads of an army of people, ranked by their SEP, with the height for each individual proportionate to the value of his/her perceived health status, expressed as a fraction of the mean for the group.

Finally, we used the framework (based on the assumption of a linear additive relationship between the health variable *y* and a set of explanatory variables *x*; i.e., *y*_*i*_ = *α* + ∑_*k*_*β*_*k*_*x*_*ki*_ + *ε*_*i*_ (*x*_*k*_ are sets of health determinants and *ε* is the disturbance term)) [51] to decompose the concentration index for *y* (perceived health) when CI is expressed as

(6) $$\begin{array}{c} CI=\underset{k}{\sum }\left(\;\frac{\beta_{K}{\overset{-}{X}}_{K}}{\mu }\;\right)C_{k}+\frac{{GC}_{\varepsilon }}{\mu }, \end{array}$$

where

 
${\overset{-}{X}}_{K}$ is the mean of *x*_*k*_;
 
*C*_*k*_ is the concentration index for *x*_*k*_ (defined analogously to *C*);
 
GC_*ε*_ is the generalized concentration index for the disturbance term.

Thus, concentration index (CI) is equal to a weighted sum of the *k* regressors. The weight for regressor *k* is the elasticity of *y* for *x*_*k*_. The residual component reflects health inequality not explained by systematic variation across SES in the regressors. The estimated health elasticity* (marginal effect)* of determinant *k* is written as ${\hat{\eta }}_{k}=({\hat{\beta }}_{K}{\overset{-}{X}}_{K}/\mu )C_{k}$, where ${\hat{\eta }}_{k}$ is the relative change of *y* statistically associated with a one-unit change of the corresponding *x*_*k*_ (a weighted average of the health levels of the sampled population when higher weights are attached to the worse-off than the better-off). Wagstaff et al. [51] argue that changing contributions can be caused either by changes in the elasticities of *η*_*k*_ or by changes in the distribution of *C*_*k*_ of *x*_*k*_.

## 3. Ethics

This study uses secondary data collected from perpetual surveys. The datasets are anonymously coded with no individual identification identifiable by the user. The users have explicit authorization to use the datasets made available for analysis.

## 4. Results

The proportion of respondents from 45 years and above age groups consistently increased in 2015 compared to 2005 and so was the representation of females in the same age groups (Table 2). Representation of rural respondents and respondents with own house was less in 2015 compared to 2010. Although the proportion of respondents with chronic disease and disability (presence of physical limitation) increased consistently from 2005 onwards, reporting of bad and very bad perceived health decreased consistently during the same period. Female respondents followed the same trend in reporting perceived health status as observed for the overall study population. In our study sample, the proportion of respondents with denial of needed healthcare attributable to the increased distance to the health facility from the respondent's residence increased consistently. The respondents from dwelling units of the not bad condition, having ownership of car and computer, with ease of survival (ability to make both ends meet), and with ease of repaying loan increased in 2015 compared to 2005. Reported neighborhood safety was found to be much better in 2015 compared to earlier years. The adult equivalent household income increased 3.6 times in 2015 from 2005 while the distance between the mean and median of the adult equivalent household income decreased by 2.5% (from 24% in 2005 to 21.5% in 2015). However, the Gini coefficient registered a positive shift by 2.2% during the study period. Our objectively determined SEP reflected a consistent increase of respondents from the poorest quintile with a consistent decrease of respondents from the richest quintile in 2015 compared to 2005. Table 2 exhibits a statistically significant association between perceived health and SEP of the respondents; average and below perceived health status increased consistently for respondents from the poorest quintile in 2015 compared to 2005.

Although the difference for total between the mean of indirectly standardized perceived health and the mean of perceived health status was found to be negative in 2015, the value of the mean of standardized variant of perceived health status was higher than that of nonstandardized variant for better-off individuals implying that inequalities were better avoided for the better-off individuals when the effects of age, gender, diagnosed chronic diseases, and presence of physical limitation (disability) were controlled (Table 3). The negative values indicated a smaller value of the mean of standardized variant of perceived health status compared to the same for nonstandardized variant reflecting that some of the inequalities in the distribution of perceived health were unavoidable and due simply to the effect of age, gender, diagnosed chronic diseases, and presence of physical limitation (disability) of the sampled population. Thus, the trend reflected consistently more unavoidable inequalities for the relatively worse-off individuals over the period. Also, the distance of standardized variant of mean perceived health status of the poorest quintiles from the standardized variant of mean perceived health status of the sampled population increased in 2015 compared to 2005. Although a negative shift of HI in 2015 compared to 2005 (Table 3) reflected a better perceived health status for the worse-off individuals, the difference in mean of nonstandardized variant of perceived health status between the richest and poorest quintiles increased during the period.

Table 4 shows the results from decomposing CI (i.e., factor level contributions to SES-related health inequalities for 2015, 2010, and 2005). A negative contribution of a factor to the CI indicated (see (6)) that the factor correlates positively with perceived health status, and such contribution is concentrated among worse-off individuals (more material deprivation); likewise, the reverse is true. The negative contribution of ownership of a house in 2015 implied that the concentration of ownership of a house among better-off individuals increased the concentration of bad and very bad perceived health among the worse-off individuals. Similarly, the probability of being employed (working status) in 2005 was associated with lower risks of bad and very bad perceived health status. The positive contribution of age in all the years moderated observed inequality; elderly respondents were vulnerable to a higher risk of having bad and very bad perceived health status even if they were members of better-off SES. The contribution of gender in the health gradient was found to be insignificant. In the decomposition of total change in the concentration index between 2005 and 2015, level of education and working status (being employed) were the most important variables in their contributions to SES-related health inequalities. There was a substantial reduction of proportional contribution of geography (in-country regional difference) in 2015 compared to 2010 (Table 4). The effects of residuals (unexplained factors) were substantially low for all the years.

## 5. Discussion

Using cross-sectional time series data from the Latvian household survey (2005–2015), we examined health inequalities in Latvia and identified the determinants of SEP producing such health inequalities in the Latvian population. While examining the distribution of perceived health status across different socioeconomic groups, we found that the overall concentration of positive perceived health favored worse-off individuals in 2015 compared to earlier years but to some extent (−0.08) inequalities in perceived health status remain unavoidable (after controlling the effect of age, gender, diagnosed chronic diseases, and presence of physical limitation) in 2015. The differences between the means of standardized and nonstandardized variants of perceived health status were consistently negative for the poorest quintile of SES while the same was consistently positive for the richest suggesting that worse-off individuals carried consistently unavoidable inequalities in perceived health during the study period. Also, the difference between the means of standardized and nonstandardized variants of perceived health status between quintiles of SES increased by 35.85% favoring the richest in 2015 compared to 2005 (difference between the richest and the poorest quintiles: 53% in 2005, 0.62 in 2010, and 0.72 in 2015). Such evidence suggested that although the health inequality index of perceived health favored the worse-off individuals, the burden of unavoidable (after controlling the effect of age, gender, diagnosed chronic diseases, and presence of physical limitation) inequalities was strong and sustained on the poorest quintile of SES. When compared to the mean perceived health status (standardized and nonstandardized variant) between the richest and the poorest quintiles of SES, we found a gradient between SEP and perceived health status, a linear decrease in health that comes with decreasing SEP. This relationship between poverty (deprivation) and poor health status is congruent with established arguments [52–54]. Such observation is also in harmony with the pathways from SES to health that shapes individual responses to perceived health status [55].

When estimating proportional contribution of different SES-related determinants in Latvian health inequalities, we found that education, household income, and ease of survival in all the years, and working status (being employed) in 2015 and 2010 were the most important factors contributing to differences in perceived health status. The high contribution of working status in 2015 compared to earlier years supported earlier findings in Latvia [11]. Such an association of no work with a higher risk for poor health is also in agreement with studies from other European countries [21, 56]. The strong and positive effects of household income in the distribution of perceived health status and the associated trend (i.e., the +ve shift of Gini index, improved material affluence, i.e., increased number of respondents in the study population from dwelling units of not bad condition, having ownership of car and computer, and with ease of survival, i.e., ability to make both ends meet and with ease of repaying loan) were consistent with the established relationship between health and SEP.

With the attempt to measure changes in the relative contributions of such determinants over the period 2005–2015, we found a substantial reduction of proportional contribution of geography (in-country regional difference) in 2015 compared to 2010. This finding in conjunction with the reduced contribution of ownership of a house can be explained with the increased shift of respondents from rural to urban settlement (Table 2) in our study population during the study period. Although the relative contributions of the factors (determinants) identified [$({\hat{\beta }}_{K}{\overset{-}{X}}_{K}/\mu )C_{k}$] registered changes in the intervening period, overall contributions (97%+) of the factors to health inequalities in perceived health status remained unchanged over the decade (2005–2015).

The findings ((1) a +ve shift of Gini index by 1.4% and an increased contribution of household income to health inequalities by 28% in 2015 from 2010 and a −ve shift of health inequality index by 11.6% during the same period, and (2) a +ve shift of Gini index by 0.8% and a decreased contribution of household income to health inequalities by 22.6% in 2010 from 2005 and a −ve shift of health inequality index by 22.9% during the same period) established the notion that income alone could not explain changes in the distribution of perceived health status. An increased shift of the respondents from rural to urban settlement is presumed to be accompanied with improved access to publicly provided services and so the changes in the distribution of perceived health status can be plausibly [57] attributed to subjective perception of relatively better rank within SES during the study period. Further, such phenomena can also be decoded as the expressions of macroeconomic factors on happiness (fluctuations in negative affect) when the economy is still dynamic, open, and volatile [58].

The strengths of this study lie in (1) using the most recent waves (2005–2015) of survey datasets to generate evidence while the economic, social, and health systems reforms are in progress, (2) unfolding the evolution of perceived health gradient for Latvians since the accession to the European Union (EU), and (3) identifying the contributing factors to inequalities in perceived health and presenting changes in the extent of such contributions to overall inequalities in perceived health over the decade of accession to the EU.

This study has few limitations as follows. (1) Despite using cross-sectional time series data for a reasonably long period, the effect of a substantial high attrition rate on perceived health status cannot be ignored. Also, cross-sectional data have the potential for reverse causation (i.e., health status affecting SEP); (2) the perceived health status variable is a bounded variable, so the use of CI is based on the assumption that the level of inequality is the same irrespective of representation (attainment versus shortfall) and so our measurement of the health inequality is not a value neutral; and (3) the decomposition method used is one-dimensional focusing perceived health (i.e., the method explains the extent of variation in perceived health instead of covariance between health and socioeconomic positions). Further, it is also true that inherent biases attributable to individual heterogeneity associated with SEP influence the perceived health status.

## 6. Conclusions

This study contributes by examining the evolution of distributional differences in perceived health status for Latvia in recent times. We conclude with the empirical evidence that (1) a favorable health inequality index does not confirm a reduced burden of unavoidable inequalities in health on the worse-off group of the population and (2) the relative contributions of SES-related determinants to the production of health inequalities change over time. Notwithstanding few explicit limitations, this study generates evidence for insightful health policy development.

## Figures and Tables

**Table 1:** Data.

Year of survey	Number of respondents	Present from the previous wave	Attrition (%) corresponding to the previous wave
2005	7913	—	
2006	9071	4258	46.18
2007	9270	4270	52.92
2008	10910	4559	50.81
2009	12207	5236	52.01
2010	12999	5743	52.95
2011	13503	6141	52.76
2012	12964	6159	54.39
2013	12442	6122	52.78
2014	11929	5927	52.36
2015	11726	5696	52.25

**Table 2:** Descriptive statistics.

Variables	2015		2010		2005	
Age group (in years)	[*N* = 11726]		[*N* = 12999]		[*N* = 7913]	
<30	16.66		21.15		22.63	
31–44	18.97		19.41		21.99	
45–60	27.16		26.98		25.15	
61–74	21.31		20.05		19.69	
75+	15.90		12.41		10.54	
Age group (in years) by gender	Female (%)		Female (%)		Female (%)	
<30	14.20		18.38		19.35	
31–44	16.96		17.92		20.51	
45–60	26.09		26.25		24.86	
61–74	23.08		21.72		21.92	
75+	19.67		15.73		13.36	
Settlement of residence (%)						
Urban	71.87		65.49		¥	
Rural	28.13		34.51		¥	
Perceived health status distribution (%)						
Very good	3.83		3.82		2.46	
Good	37.14		39.67		30.54	
Average	40.06		36.77		43.62	
Bad	15.21		15.81		17.33	
Very bad	3.76		3.93		6.05	
Perceived health status (%) (gender = female)						
Very good	2.99		3.03		1.64	
Good	33.97		36.30		26.94	
Average	41.50		38.55		44.32	
Bad	17.07		17.60		19.91	
Very bad	4.47		4.52		7.19	
Denial of needed healthcare services Reason (% of denied services)	12.36		21.35		29.66	
Affordability	59.48		63.32		57.43	
Distance	3.32		2.76		2.09	
Work/childcare	5.87		7.31		11.74	
Chronic disease (%)	46.01		38.87		37.72	
Disability (%)	42.58		34.57		34.66	
Working status (%)						
Employed	49.31		41.85		50.12	
Retired	31.20		29.99		29.42	
Ownership of house (%)	81.72		85.41		78.91	
Overall condition of dwelling unit as bad (%)	24.92		25.19		39.90	
Vulnerable neighborhood security/safety (%)	11.69		22.40		21.82	
Ownership of car (%)	55.12		51.43		42.61	
Ownership of computer (%)	71.72		59.64		33.67	
Ease of survival; ability to make both ends meet (%), with difficulty	78.49		85.48		86.98	
Ease of paying the loan (%), with difficulty	72.52		82.47		79.81	
Adult equivalent household income (€)						
Mean	11627.90		8899.24		3197.83	
Median	9570.68		7195.88		2578.24	
Gini coefficient	0.365		0.360		0.357	
Socioeconomic position (SEP) distribution (%)						
Poorest	24.00		22.12		21.36	
2nd poorest	21.07		21.47		21.13	
Middle	19.17		19.89		19.94	
2nd richest	18.54		18.36		19.34	
Richest	17.22		18.16		18.23	
Distribution of perceived health (%) across SEP	Average	Bad and very bad	Average	Bad and very bad	Average	Bad and very bad
Poorest	26.13	52.21	24.32	43.88	18.82	39.30
2nd poorest	23.65	23.50	23.43	27.94	20.74	29.86
Middle	19.04	13.13	21.14	14.09	21.93	15.77
2nd richest	17.63	7.93	16.84	9.00	19.92	10.57
Richest	13.55	3.23	14.28	5.09	18.59	4.50
Chi-square (*χ*^2^)	0.000		0.000		0.000	

¥: no data of the variable is available in the wave.

**Table 3:** Distribution of perceived health status.

SES quintile	2015 [*N* = 11726]			2010 [*N* = 12999]			2005 [*N* = 7913]		
	Δ	^∧^Mean-std.	^∧∧^Mean	Δ	^∧^Mean-std.	^∧∧^Mean	Δ	^∧^Mean-std.	^∧∧^Mean
Poorest	−0.51	2.88	3.38	−0.32	2.94	3.26	−0.15	3.13	3.27
2nd poorest	−0.35	2.76	3.11	−0.11	2.86	2.97	0.08	3.00	2.93
Middle	−0.08	2.69	2.78	0.12	2.80	2.68	0.24	2.92	2.67
2nd richest	0.08	2.68	2.60	0.25	2.75	2.51	0.31	2.87	2.57
Richest	0.21	2.60	2.40	0.30	2.65	2.35	0.38	2.77	2.39
Total	−0.08	2.70	2.78	0.04	2.80	2.76	0.07	3.01	2.94
HI	0.137 (0.004)			0.155 (0.004)			0.201 (0.006)		

^∧^Mean of indirectly standardized perceived health status; ^∧∧^mean of perceived health status (1 = very good, 2 = good, 3 = average, 4 = bad, and 5 = very bad); Δ: difference between the mean of indirectly standardized perceived health and the mean of perceived health status; HI: health inequality index. Figures in parentheses indicate bootstrapped standard error.

**Table 4:** Factors contributing to health inequalities.

Effects and contributions of predictor variables	Change in contribution (%) 2005–2015	2015 [*N* = 11726]		2010 [*N* = 12999]		2005 [*N* = 7913]	
		Marginal effect	% contribution	Marginal effect	% contribution	Marginal effect	% contribution
Age	15.9	−0.33	25.70	−0.54	29.10	−0.71	41.60
Gender (=female)	2.0	0.01	−0.50	−0.00	0.10	−0.04	1.50
Education	−2.6	0.03	9.70	0.02	10.00	0.03	7.10
Working status	−14.0	0.04	10.90	0.02	5.80	−0.01	−3.10
Household income	0.4	0.71	43.90	0.49	34.30	0.88	44.30
Ease of survival	1.2	0.01	7.20	0.02	9.80	0.02	8.40
Ownership of house	0.5	−0.01	−0.40	0.01	0.60	0.0	0.10
Regional effect			0.30		7.30		¥
Residual		0.002		0.002		−0.001	

¥: no data for the variable is available in the wave.

## References

[1] Kawachi I., Berkman and L., Kawachi I. (2000). Income Inequality and Health. *Social Epidemiology*.

[2] Lynch J. W., Smith G. D., Kaplan G. A., House J. S. (2000). Income inequality and mortality: Importance to health of individual income, psychosocial environment, or material conditions. *British Medical Journal*.

[3] Lynch J. W., Kaplan G. A., Pamuk E. R. (1998). Income inequality and mortality in metropolitan areas of the United States. *American Journal of Public Health*.

[4] Lynch J. W., Kaplan G. A., Salonen J. T. (1997). Why do poor people behave poorly? Variation in adult health behaviours and psychosocial characteristics by stages of the socioeconomic lifecourse. *Social Science and Medicine*.

[5] Wilkinson R. (1996). *Unhealthy societies: The Afflictions of Inequality*.

[6] Kaplan G. A. (1996). People and places: Contrasting perspectives on the association between social class and health. *International Journal of Health Services*.

[7] Kennedy B. P., Kawachi I., Prothrow-Stith D. (1996). Income distribution and mortality: Cross sectional ecological study of the Robin Hood index in the United States. *British Medical Journal*.

[8] Link B. G., Phelan J. C. (2002). Mckeown and the idea that social conditions are fundamental causes of disease. *American Journal of Public Health*.

[9] Link B. G., Phelan J. (1995). Social conditions as fundamental causes of disease. *Journal of Health and Social Behavior*.

[10] Lochner K., Pamuk E., Makuc D., Kennedy B. P., Kawachi I. (2001). State-level income inequality and individual mortality risk: A prospective, multilevel study. *American Journal of Public Health*.

[11] Monden C. W. S. (2004). Socioeconomic health inequalities in Latvia: A cross-sectional study. *Scandinavian Journal of Public Health*.

[12] Gilson L. (1998). In defence and pursuit of equity. *Social Science and Medicine*.

[13] Wilkinson R. G. (1997). Socioeconomic determinants of health: Health inequalities: Relative or absolute material standards?. *British Medical Journal*.

[14] Manor O., Matthews S., Power C. (1997). Comparing measures of health inequality. *Social Science and Medicine*.

[15] Mcisaac S. J., Wilkinson R. G. (1997). Income distribution and cause-specific mortality. *The European Journal of Public Health*.

[16] Kunst A. E., Mackenbach J. P. (1994). The size of mortality differences associated with educational level in nine industrialized countries. *American Journal of Public Health*.

[17] Blaxter M. A. (1989). Comparison of measures of inequality in morbidity. *Health Inequalities in European Countries*.

[18] Jones K., Moon G. (1987). *Health, disease, and society: a critical medical geography , Routledge and Kegan*.

[19] Marmot M., Ryff C. D., Bumpass L. L., Shipley M., Marks N. F. (1997). Social inequalities in health: Next questions and converging evidence. *Social Science and Medicine*.

[20] Eikemo T. A., Bambra C., Judge K., Ringdal K. (2008). Welfare state regimes and differences in self-perceived health in Europe: A multilevel analysis. *Social Science and Medicine*.

[21] Eikemo T. A., Huisman M., Bambra C., Kunst A. E. (2008). Health inequalities according to educational level in different welfare regimes: A comparison of 23 European countries. *Sociology of Health and Illness*.

[22] Rodgers G. B., Kawachi I., Kennedy B. P., Wilkinson R. (1999). Income and inequality as determinants of mortality. *The Society and Population Health Reader*.

[23] Coburn D. (2004). Beyond the income inequality hypothesis: Class, neo-liberalism, and health inequalities. *Social Science and Medicine*.

[24] Komlos J. (1999). On the Biological Standard of Living in Russia and the Soviet Union. *Slavic Review*.

[25] Steckel R. (1995). Stature and the standard of living. *J. Econ. Lit*.

[26] Gwatkin D. (2000). Health inequalities and the health of the poor: what do we know? What can we do?. *Bulletin of the World Health Organization*.

[27] Bobak M., Pikhart H., Rose R., Hertzman C., Marmot M. (2000). Socioeconomic factors, material inequalities, and perceived control in self-rated health: Cross-sectional data from seven post-communist countries. *Social Science and Medicine*.

[28] Carlson P. (1998). Self-perceived health in East and West Europe: Another European health divide. *Social Science and Medicine*.

[29] Monden C. W. S. (2005). Changing social variations in self-assessed health in times of transition? The Baltic States 1994-1999. *European Journal of Public Health*.

[30] Leinsalu M. (2002). Social variation in self-rated health in Estonia: A cross-sectional study. *Social Science and Medicine*.

[31] Kalediene R., Petrauskiene J. (2000). Inequalities in life expectancy in Lithuania by level of education. *Scandinavian Journal of Public Health*.

[32] UNDP (1999). *Human development and conflict in simultaneously occurring processes of modernisation and post-modernisation*.

[33] Ministry of Economy., Economic Development of Latvia ., Riga, Ministry of Economy,

[34] UNDP (1997). *Latvia human development report*.

[35] Wamala S. P., Lynch J., Kaplan G. A. (2001). Women's exposure to early and later life socioeconomic disadvantage and coronary heart disease risk: The Stockholm Female Coronary Risk Study. *International Journal of Epidemiology*.

[36] Paul P., Valtonen H. (2016). Health inequality in the Russian Federation: An examination of the changes in concentration and achievement indices from 1994 to 2013. *International Journal for Equity in Health*.

[37] Možajeva I. Multidimensional Health Modeling: Association Between Socioeconomic and Psychosocial Factors and Health in Latvia. *he Economic Research Guardian*.

[38] Mackenbach J. P., Stirbu I., Roskam A.-J. R. (2008). Socioeconomic inequalities in health in 22 European countries. *New England Journal of Medicine*.

[39] Adams P., Hurd M. D., McFadden D., Merrill A., Ribeiro T. (2003). Healthy, wealthy, and wise? Tests for direct causal paths between health and socioeconomic status. *Journal of Econometrics*.

[40] Frijters P., Haisken-DeNew J. P., Shields M. A. (2003). Estimating the causal effect of income on health: evidence from postreunification East Germany. *Centre for Economic Policy Discussion*.

[41] Benzeval M., Judge K., Shouls S. (2001). Understanding the relationship between income and health: How much can be gleaned from cross-sectional data?. *Social Policy and Administration*.

[42] Smith J. P. (1999). Healthy bodies and thick wallets: The dual relation between health and economic status. *Journal of Economic Perspectives*.

[43] McKenzie D. J. (2003). Measure inequality with asset indicators. *BREAD Working Paper*.

[44] Schokkaert E., Van De Voorde C. (2004). Risk selection and the specification of the conventional risk adjustment formula. *Journal of Health Economics*.

[45] Gravelle H. (2003). Measuring income related inequality in health: Standardisation and the partial concentration index. *Health Economics*.

[46] Benyamini Y., Idler E. L. (1999). Community studies reporting association between self-rated health and mortality: additional studies, 1995 to 1998. *Research on Aging*.

[47] Idler E. L., Benyamini Y. (1997). Self-rated health and mortality: a review of twenty-seven community studies. *Journal of Health and Social Behavior*.

[48] Wolinsky Fredric D., Tierey William M. (1998). Self-rated health and adverse health outcomes: An exploration and refinement of the trajectory hypothesis. *Journal of Gerontology: Social Sciences*.

[49] Wagstaff A. (2005). The bounds of the concentration index when the variable of interest is binary, with an application to immunization inequality. *Health Economics*.

[50] Kakwani N., Wagstaff A., Van Doorslaer E. (1997). Socioeconomic inequalities in health: Measurement, computation, and statistical inference. *Journal of Econometrics*.

[51] Wagstaff A., van Doorslaer E., Watanabe N. (2003). On decomposing the causes of health sector inequalities with an application to malnutrition inequalities in Vietnam. *Journal of Econometrics*.

[52] Marmot M. (2004). The status syndrome: How social status affects our health and longevity. *Times*.

[53] Klugman J. (1997). Poverty in Russia: public policy and private responses. *The World Bank*.

[54] Alvarez-Galvez J., Rodero-Cosano M. L., Motrico E., Salinas-Perez J. A., Garcia-Alonso C., Salvador-Carulla L. (2013). The impact of socio-economic status on self-rated health: Study of 29 countries using European social surveys (2002-2008). *International Journal of Environmental Research and Public Health*.

[55] Adler N. E., Ostrove J. M. (1999). Socioeconomic status and health: What we know and what we don't. *Annals of the New York Academy of Sciences*.

[56] van Doorslaer E., Koolman X. (2004). Explaining the differences in income-related health inequalities across European countries. *Health Economics*.

[57] Adler N. E., Epel E. S., Castellazzo G., Ickovics J. R. (2000). Relationship of subjective and objective social status with psychological and physiological functioning: Preliminary data in healthy white women. *Health Psychology*.

[58] Rodríguez-Pose A., Maslauskaite K. (2012). Can policy make us happier? Individual characteristics, socio-economic factors and life satisfaction in Central and Eastern Europe. *Cambridge Journal of Regions, Economy and Society*.

